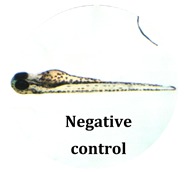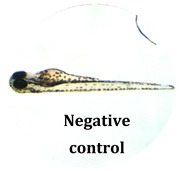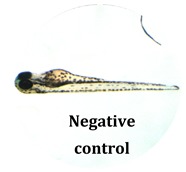# Flavonoid Analysis and Antioxidant Activities of the *Bryonia alba* L. Aerial Parts

**DOI:** 10.3390/antiox8040108

**Published:** 2019-04-20

**Authors:** Irina Ielciu, Michel Frédérich, Daniela Hanganu, Luc Angenot, Neli-Kinga Olah, Allison Ledoux, Gianina Crișan, Ramona Păltinean

**Affiliations:** 1 Department of Pharmaceutical Botany, Faculty of Pharmacy, “Iuliu Hațieganu” University of Medicine and Pharmacy, 400337 Cluj-Napoca, Romania; irina.ielciu@umfcluj.ro (I.I.); gcrisan@umfcluj.ro (G.C.); rpaltinean@umfcluj.ro (R.P.); 2 Laboratory of Pharmacognosy, Center of Interdisciplinary Research on Medicines, University of Liège, 4000 Liège, Belgium; M.Frederich@uliege.be (M.F.); l.angenot@uliege.be (L.A.); allison.ledoux@uliege.be (A.L.); 3 Department of Pharmacognosy, Faculty of Pharmacy, “Iuliu Haţieganu” University of Medicine and Pharmacy, 400010 Cluj-Napoca, Romania; 4 PlantExtrakt Ltd., Rădaia, 407059 Cluj-Napoca, Romania; neliolah@yahoo.com; 5 Department of Pharmaceutical Industry, Faculty of Pharmacy, "Vasile Goldiş" Western University of Arad, 310045 Arad, Romania

**Keywords:** flavonoids, HPLC-DAD, antioxidant, toxicity

## Abstract

*Bryonia alba* L. is the only *Bryonia* species found in Romanian flora, being known as a remedy for inflammatory pathologies or for its hepatoprotective and adaptogen activities. The present investigation studied the flavonoid composition and antioxidant activities of the aerial parts of this species. Flavonoid profile was evaluated by HPLC coupled with Diode Array Detection (DAD), while antioxidant capacity was assessed by various methods, testing different antioxidant mechanisms: DPPH (2,2-diphenyl-1-picrylhydrazyl), CUPRAC (cupric reducing antioxidant capacity), FRAP (ferric reducing ability of plasma), TEAC (Trolox equivalent antioxidant capacity), EPR (electron paramagnetic resonance method) and SNPAC (silver nanoparticles antioxidant capacity). Cytotoxicity was tested on human cancerous and healthy cell lines. Anti-plasmodial tests were performed on two strains of *Plasmodium falciparum*. Whole organism toxicity was assessed on zebrafish larvae. The HPLC-DAD analysis proved the presence of lutonarin, saponarin, isoorientin, and isovitexin as the major flavonoids in the composition of tested samples. Significant results were obtained for all antioxidant capacity assays. The cytotoxicity tests proved the absence of cellular and parasitic toxicity and these results were confirmed by the lack of toxicity on the zebrafish larvae model. This study proves a promising potential of the aerial parts of *Bryonia alba* L. as antioxidant agents.

## 1. Introduction

The Cucurbitales order is a taxonomic unit with worldwide distribution, having significant diversity in species. It comprises almost 2600 species, contains around 110 genera, and it is divided in seven families, mostly found in temperate climates: Anisophylaceae, Begoniaceae, Coriariaceae, Corynocarpaceae, Cucurbitaceae, Datisticaceae, and Tetramelaceae [1,2]. Among these families, the Cucurbitaceae family is the largest, comprising over 100 genera and 800 species [1], mostly found in tropical and subtropical regions, in south-eastern Asia, western Africa, Madagascar, and Mexico [1,3,4]. Cucurbitaceae species can also be found naturalized in Europe, in different areas, especially in the southern, central and eastern parts of the continent. Because a significant number of species are used for economic purposes, their cultivation is highly spread, especially in southern Europe [3]. 

Among the Cucurbitaceae genera found in Europe, *Bryonia* is one of the largest, comprising 10 species, distributed from the Mediterranean to Central European region [3], Northern Africa and Central Asia [1,5]. Kocyan et al. [5] included this genus in the Cucurbitoideae subfamily, Bryonieae tribe, which also includes, together with the 10 *Bryonia* species, the *Ecballium elaterium* (L.) A. Rich. species [5,6]. Relationships between the two genera are related to morphological characteristics and to medicinal uses. Differences especially concern the breeding system, as *E. elaterium* comprises two subspecies with different breeding systems (subspecies *dioicum*, which is dioecious, and subspecies *elaterium*, which is monoecious), while in the *Bryonia* genus seven species are dioecious and three are monoecious [7,8]. 

In the Romanian flora, *Bryonia* has been the only genus of the Cucurbitaceae family that created confusion among botanists. The presence of two species is cited: *B. alba* L. and *B. dioica* Jacq., but confusions are especially related to the latter. Tutin et al., in *Flora Europaea*, cite the presence of one species, *Bryonia cretica* L., with three subspecies, namely *cretica*, *dioica*, and *acuta* [3], including the species *B. dioica* as a subspecies of *B. cretica*. Romanian sources cite the presence of *Bryonia dioica* Jacq. [9,10], whilst the most recent sources regarding Romanian flora cite the presence of *Bryonia cretica* L. subsp. *dioica* [11], probably following the classification of *Flora Europaea*. The presence of *B. dioica* in the Romanian country remains uncertain, as it is recorded as doubtful by Săvulescu et al. in an older Romanian flora [9]. In a more recent flora of Romania, Ciocârlan et al. describe the presence of the species in Arad, Radna, and Bucharest [10]. Studies performed by Volz and Renner in 2008 and 2009 [6,12] cite its presence as uncertain, stating that it might have escaped from the Botanical Garden in Bucharest, Romania. 

Confusions between these species are due to the large number of similarities between them. However, differences can also be found. The most significant difference between these species, besides morphological characteristics, is found in the breeding system, as *B. alba* L. is a monoecious species, while *B. cretica* L. and *B. dioica* Jacq. are dioecious species. Distribution of these species is also different: *B. alba* ranges from Central Europe to Kazakhstan, while *B. cretica* L. and *B. dioica* have different area distributions, especially the southern, central, and eastern parts of Europe, where *B. dioica* appears to be spreading. Distribution of the major chloroplast haplotypes for *Bryonia* species, from a geographic point of view, suggest that *B. alba* L. is highly likely to represent the only *Bryonia* species in Romania and the most widespread *Bryonia* species in the world [12]. 

In this context, and taking into consideration that *B. alba* is the only species belonging to the *Bryonia* genus that has been reported in Romania, it appears necessary to study its chemical composition and biological activity in detail, especially as it is reported to have therapeutic uses [7,8]. Not only is this species reported to have important homeopathic uses, but it is also reported to have traditional uses as antipyretic, anti-inflammatory, antibacterial, laxative-purgative, smooth muscle relaxant, cytotoxic, hepatoprotective, and anti-diabetic properties [8], which prove its promising potential for the treatment of various diseases. 

The present study was performed in the context of similar studies on this species, and other related species belonging to Cucurbitaceae family, that have aimed to reveal the antioxidant potential of some species belonging to this family that are lesser known for their biological activity or economical uses [13,14,15]. At the same time, the aim of this study was to reveal the fact that *Bryonia alba* lacks toxicity in all its aerial parts, and to prove its promising potential as an antioxidant agent. The novelty of the study lies in its testing the lack of toxicity on different in vitro models, namely human cancerous cell lines, healthy cell lines, and parasitic strains, and on one in vivo model, the zebrafish larvae model. The reason for choosing these models lies in the antioxidant activity that was further tested by several non-cellular assays, which can boost a possible antiplasmodial activity. The present study appears in the context of several antioxidant molecules that have been proven to have significant antioxidant activity, showing other important biological activities besides anti-plasmodial (e.g., ellagic acid) [16]. Therefore, the aim of this study was to assess the flavonoid composition of the aerial parts of *Bryonia alba* L., and to evaluate their antioxidant potential, proving at the same time that these parts lack cellular toxicity both in vitro and in vivo.

## 2. Materials and Methods 

### 2.1. Plant Material

Aerial parts of *Bryonia alba* L. were collected from Cluj-Napoca county in July 2014 (north-western Romania) during the flowering period. The vegetal material was identified at the Department of Pharmaceutical Botany of the “Iuliu Haţieganu” University of Medicine and Pharmacy (Cluj-Napoca, Romania), where voucher specimens nr. 105.3.1.1–12 were deposited in the Herbarium of the Department. 

### 2.2. Extraction Procedure

The collected vegetal material was ground in order to obtain a fine powder. Vegetal powder (50 g) was macerated with 500 mL methanol for 24 h. Subsequently, powder was subjected to percolation and the obtained solution was evaporated under vacuum at 40 °C. The crude extract obtained was subjected to the analysis of compounds by HPLC coupled with Diode Array Detection (HPLC-DAD) analysis and to evaluation of antioxidant capacity after being dissolved in analytical grade ethanol (Merck, Darmstadt, Germany). Samples that were subjected to cytotoxicity assays were obtained by dissolving crude extracts in dimethyl sulfoxide (DMSO, Merck, Darmstadt, Germany) at a concentration of 10 mg/mL, while for whole organism toxicity test on zebrafish, crude extracts were suspended in the water used for the development of zebrafish larvae (concentration 1 mg/mL) [15]. 

### 2.3. Chemicals

Folin-Ciocâlteu reagent, sodium carbonate, gallic acid, aluminium chloride, sodium acetate, and rutin were purchased from Sigma-Aldrich (Darmstadt, Germany). Flavonoid references used for HPLC-DAD analysis (saponarin—purity ≥ 98%, isoorientin—purity ≥ 99%, and isovitexin—purity ≥ 99%) were obtained from Extrasynthèse (Lyon, France). Human cancerous cell lines (HeLa, A549) and human healthy cell line (WI-38) were obtained from the Laboratory of Medical Chemistry (GIGA Center, University of Liège, Liège, Belgium). All reagents used for cell culture assays were purchased from Bio-Whittaker-Lonza (Verviers, Belgium). Cell proliferation reagent (WST-1) was purchased from Roche (Bâle, Switzerland). Reagents for antioxidant assays were purchased from Sigma-Aldrich (Darmstadt, Germany). 

### 2.4. Determination of Total Phenolic Content (TPC) 

The total phenolic content (TPC) was evaluated by a method that uses the Folin-Ciocâlteu reagent, with some changes compared to the classical one. Methanolic extract (2.0 mL) was diluted 25 times and then mixed with 1.0 mL Folin-Ciocâlteu reagent and 10.0 mL of distilled water, and diluted to 25.0 mL with 290 g/L sodium carbonate. After 30 min incubation in the dark, absorbance of the solution was determined at 760 nm. Results were expressed as mg gallic acid equivalents (GAE)/100 g dry vegetal product (dvp), using a calibration curve based on gallic acid (*R*^2^ = 0.998). Spectrophotometric data were acquired using a Jasco V-530 UV-Vis spectrophotometer (Jasco International Co., Ltd., Tokyo, Japan) [17].

### 2.5. Determination of Total Flavonoid Content (TFC)

The total flavonoid content (TFC) was assessed by a spectrophotometric method based on the formation of the aluminum chloride complex. Methanolic extract (5.0 mL) was mixed with 5.0 mL sodium acetate 100 g/L, 3.0 mL aluminum chloride 25 g/L and completed to 25.0 mL in a calibrated flask. Absorbance of the resulted solution was measured at 430 nm. Results were expressed as mg rutin equivalents (RE)/100 g dvp, using a calibration curve based on rutin (*R*^2^ = 0.999) [17,18].

### 2.6. Identification and Quantification of Flavonoids 

#### HPLC-DAD Analysis of Flavonoids

Flavonoids were analyzed by a method described by Ielciu et al. [15]. Crude extracts dissolved in methanol (1 mg/mL) were filtered through a 0.45 mm membrane and injected into a Hewlett Packard Agilent 1100 system (Agilent, Santa Clara, California, USA), equipped with an Agilent 1100 quaternary pump (Agilent, Santa Clara, California, USA), an Agilent 1100 degaser, and an Agilent 1100 Automatic Liquid Sampler (Agilent, Santa Clara, California, USA ). Mobile phases were represented by 0.05% *V/V* triflouroacetic acid in water (A) and acetonitrile (B), at a flow rate of 1 mL/min, with the following gradient composition: 0 to 1 min 0% B; 1 to 45 min 3% B; 45 to 55 min 40% B; 55 to 56 min 60% B; 56 to 66 min 60% B; 66 to 67 min 0% B; and 67 to 82 min 0% B. For separation, a Hypersil ODS C18 column (250 mm × 4.6, i.d.; particle size 5μm) was used. Temperature of separation was set at 25 °C. Detection of flavonoids was performed at 350 nm on an Agilent 1200 Diode Array Detector (Agilent, Santa Clara, California, USA). In order to identify the flavonoids, comparison of retention times and UV-VIS spectra of reference compounds was performed.

### 2.7. Antioxidant Activity Assays

#### 2.7.1. DPPH Assay

The classical DPPH method is based on the neutralization of the 2,2-diphenyl-1-picrylhydrazyl (DPPH) free radical, which changes the color from violet to yellow in the presence of an antioxidant. The color change is correlated with the antioxidant capacity, and is evaluated by spectrophotometry at 517 nm. A mixture of 5 mL of DPPH (25 mM) and 5 mL of sample was incubated for 30 min at 40 °C. The reference solution consisted in a mixture of 5 mL of DPPH and 5 mL methanol, and the blank consisted of methanol. Results were expressed as IC_50_ (μg/mL), whichrepresents the concentration of extract that is necessary to cause 50% inhibition of the radical. The following formula was used in order to determine the inhibition percentage: I% = (Ar – As) × 100/Ar, where Ar is the absorbance of the reference solution and As is the absorbance of the sample. Quercetine and butyl-hydroxytoluene (BHT) were used as positive controls [19,20].

#### 2.7.2. Trolox Equivalents Antioxidant Capacity (TEAC) Assay

The assay evaluates the capacity of an antioxidant to reduce the 2,2-azino-bis-(3-ethyl-benzothiazoline-6-sulfonic acid) free cationic radical (ABTS•^+^) by adding a solution of potassium persulfate. The method assesses by spectrophotometry the color change of the initial solution from blue to yellow or colorless. To 1 mL of 7.5 mM ABTS solution, 1 mL of 2.6 mM potassium persulfate solution was added and the mixture was incubated for 12 h in the dark. Methanol (60.0 mL) was then added to 1 mL of this mixture (ABTS reagent). At 1 mL of sample, the ABTS solution was added and incubated in the dark for 2 h. The color change was assessed by a spectrometric determination of the absorbance at 734 nm. The reference solution consisted of the same mixture using methanol instead of the samples. The blank consisted of methanol. Results are expressed as IC_50_ (μg/mL), compared to a Trolox standard. The same formula as in the case of DPPH was used to calculate the inhibition percentage [19,21,22]. 

#### 2.7.3. CUPRAC (Cupric Reducing Antioxidant Capacity) Assay

This assay evaluates the change of color determined by the reduction of the copper ion II to the copper ion I, which takes place in the complex 2,9-dimethyl-1,10-phenantroline (neocupreine). The color change from light green to orange is correlated with the concentration of the antioxidant, and the absorbance which is correlated was measured at 450 nm. The CUPRAC reagent consisted of a mixture of 1 mL 7.5 mM neocupreine solution, 1 mL 10 mM copper chloride solution, and 1 mL ammonium acetate buffer at pH = 6.8. At 1 mL of sample, the CUPRAC reagent was added, and after incubation for 30 minutes at room temperature, absorbance was measured. The blank consisted of a solution that contained water instead of the sample. Results were expressed as mM Trolox equivalents/100 mL extract, using a calibration curve plotted with different concentration of Trolox [20,23].

#### 2.7.4. FRAP (Ferric Reducing Ability of Plasma) Assay

The ion reduced in this assay is iron, which is reduced from the ferric ion to the ferrous ion in a complex of iron with the radical 2,4,6-tripyridyl-s-triazine (TPTZ). Reduction of this ion is assessed by measuring the absorbance determined by the color change from green to yellow or blue, at 593 nm. The FRAP reagent consists of a mixture of 2.5 ml 10 mM TPTZ solution in 40 mM hydrochloric acid, to which 2.5 ml 20 mM ferric chloride solution and 25 ml acetate buffer at pH = 3.6 was added. At 1 mL of sample, 6 mL of the FRAP reagent was added and absorbance was measured. The blank consisted of a solution that contained water instead of the sample. Results are expressed as mM Trolox equivalents/100 mL extract, using a calibration curve plotted with concentrations of a Trolox standard [19,20,24]. 

#### 2.7.5. SNPAC (Silver Nanoparticles Antioxidant Capacity) Assay

The basis of this assay is represented by the silver nanoparticles (SNP) that are obtained by reducing silver ions from silver nitrate, using as surface stabilizer trisodium citrate. The silver ion is reduced to colloidal silver, using the SNP suspended in solution, by the potential antioxidants. The color change from yellow to brown is assessed by measuring the absorbance at 423 nm, using the calibration curve plotted using the Trolox standard. At 2 mL of SNP reagent obtained from 10 mM silver nitrate solution and 1% trisodium citrate solution at 100 °C, 1 mL of the sample was added. The mixture was incubated at room temperature for 30 min. The blank consisted of a solution that contained water instead of samples. Results are expressed as mM Trolox equivalents/100 mL extract [18,23].

#### 2.7.6. EPR (Electron Paramagnetic Resonance) Assay

Using the DPPH radical added to the extract, this method records the EPR spectra of the mixture. Measurements were performed on a Bruker Elexsys E500 spectrometer (Bruker, Billerica, MA, USA), in EPR quartz capillaries. The difference between samples and the DPPH radical were expressed as integral intensity (I), which was compared to that of a 4.5 mM DPPH reference, mixed with 10 μL of extract and transferred into the EPR quartz capillaries. Measurements were performed in X band (~9.4 GHz), at room temperature, with a frequency modulation of 100 kHz. Parameters used for the evaluation of samples were: center field 3360 G; sweep width 60 G; power 2 mW; receiver gain 1 × 103; modulation amplitude 2 G; time of conversion 15 ms; time constant 30.72 ms; sweep time 60s. EPR spectra were recorded at different time intervals. Variations of the relative concentration of paramagnetic species were obtained through double integration of experimental spectra using XEPR Bruker software Bruker, Billerica, USA) [18,23,25].

### 2.8. Cytotoxicity Assays

The cell lines used to test the cytotoxicity were the A549 (lung cancer), HeLa (cervical cancer), and WI38 (fetal lung fibroblasts) cell lines. Each of these cell lines was cultured in DMEM medium, supplemented with 10% heat-inactivated fetal bovine serum (FBS), 1% L-glutamine (200 mM), and penicillin (100 IU/ mL)−streptomycin (100 μg/mL), as described by Ledoux et al. [26]. A total 8000 cells were seeded per well in microplates. After 24 h, dilutions of the samples were added to each well and incubated together for 48 h. Concentrations of samples ranged between 2 and 100 μg/mL. Cell viability was assessed after 48 h by adding WST-1 tetrazolium salt and measuring absorbances at 450 nm on a Multiwell Scanner (Stat Fax 2100, Awareness Technology, Perchtoldsdorf, Austria). Samples were tested in triplicate, and tests were performed twice. For each sample, IC_50_ was calculated by linear regression, by comparison with the negative control (non-treated cells). Camptothecin was used as the positive reference [26,27]. 

### 2.9. Anti-Plasmodial Assays

Assays were performed on two *Plasmodium falciparum* strains: *3D7* (chloroquine sensitive) and *W2* (chloroquine resistant). Eight two-fold dilutions of samples in culture medium were incubated with a parasitic suspension for 48 h in 96 well microplates. Tested concentrations of samples ranged between 0.8 and 100 μg/mL. Inhibition of parasite growth was evaluated by spectrophotometry, by assessing the activity of the plasmodial lactate dehydrogenase (pLDH) at 630 nm. Absorbances were determined using a Multiwell scanner (Stat Fax 2100, Awareness Technology Inc). Positive reference for the inhibition of parasite growth was represented by artemisinin (Sigma-Aldrich), while positive and negative controls were represented by infected and uninfected erythrocytes. Inhibition of parasite growth was evaluated by comparison with non-treated erythrocytes infected with parasites, which represent 100% growth. IC_50_ values were calculated by linear regression from the concentrations range of samples. Each sample was tested two times in each test, and tests were performed in triplicate for each strain [26,28]. 

### 2.10. Zebrafish Toxicity Assays

Zebrafish (*Danio rerio*) were maintained in special conditions, according to the criteria of the Ethical Committee for the Use of Laboratory Animals (University of Liège, Liège, Belgium). Fertilized eggs were incubated at 28 °C and maintained on a 14 h day/10 h night period throughout the experiment. Larvae were collected and their chorions were removed. The larvae medium containing the crude extract (concentrations between 0.1 and 100 μg/mL) was replaced once per day. Embryos were observed each day until 72 hpf (hours post fertilization). Twenty-five larvae were treated once daily with 5 mL of each sample concentration, and another 25 were used as control [15,26]. 

### 2.11. Statistical Analysis

Data are presented as mean values ± standard deviation (SD). For each assay, samples were analyzed in triplicate. Averages and SD were calculated using the Excel software package [13,14].

## 3. Results and Discussion

### 3.1. Identification and Quantification of Flavonoids by HPLC-DAD

In the crude extract obtained from the aerial parts of *Bryonia alba* L., four flavonoids were identified: lutonarin, saponarin, isoorientin, and isovitexin, as seen in Table 1. Identification of these compounds was performed by comparison with commercially available references, which were analyzed in the same chromatographic conditions. For saponarin, isoorientin, and isovitexin, identification was achieved by comparing UV spectra of the existing references, while for lutonarin, identification was achieved by MS and NMR RMN. Previous reports exist for the presence of lutonarin, saponarin, and isovitexin [29,30] in the aerial parts of this species, while this is the first report for isoorientin, its presence being confirmed only in studies performed on the leaves of the species [15]. 

### 3.2. Determination of Total Phenolic Content (TPC) and Total Flavonoid Content (TFC)

Results obtained for the quantification of the TPC and TFC can be found in Table 2, and can be correlated with the results obtained in the biological assays, especially in the assays used to evaluate the antioxidant capacity. This is the first report that quantifies the TPC and TFC for the aerial parts of this species. Other studies performed by our team report similar amounts of both types of compounds for a species belonging to Cucurbitaceae family, *Echinocystis lobata* Torr. et A. Gray, using similar methods and similar type of samples. If comparing the results obtained for the quantification of these compounds in the aerial parts of *E. lobata* to the ones obtained for the quantification of these compounds in the aerial parts of *B. alba*, similar amounts appear to be found in all tested samples. For *E. lobata*, it appears that the TPC and TFC increase as the species reaches its maximal development period. For *B. alba*, amount of these compounds was close to the ones obtained at the maximal development period of *E. lobata*, which is in strong correlation with the collecting time, July, when this species reaches its highest peak in the accumulation of these compounds [13].

### 3.3. Antioxidant Activity Assays

Six in vitro assays were used to evaluate the antioxidant capacity of the aerial parts of *Bryonia alba*. Each of the assays performed provided different results, but connections between these results and the flavonoid analyses could be established. Results obtained for the assessment of the antioxidant capacity can be found in Table 3. It can be observed that the aerial parts of the species presented significant antioxidant capacity. This is the first report that evaluates the antioxidant capacity of the aerial parts of this species, antioxidant activity having been reported by other mechanisms and type of assays, using cellular approaches, for the leaves of the species [15].

It can be noticed that results obtained for the quantification of TPC and TFC are strongly correlated to the results obtained in the antioxidant activity assays. Similar research performed by our team on the flowers, leaves, and aerial parts of *Echinocystis lobata* Torr. et A. Gray, also belonging to the Cucurbitaceae family, reveal that the antioxidant potential of the aerial parts of *Bryonia alba* L. is important and superior to that of *E. lobata* [13,14]. Results obtained for the same assays in the evaluation of the antioxidant capacity of the aerial parts of *E. lobata* show that, for the all the assays that were used, the potential of the *B. alba* aerial parts is at least similar to that obtained for *E. lobata*. The study performed for *E. lobata* compared the antioxidant potential of this species for samples collected at the beginning and towards the maximal development period of this species. The samples that were tested in the present study were collected in the maximal accumulation period of active compounds for *B. alba*, and it is clear by comparison that their antioxidant potential is similar. For *E. lobata*, results show values of IC_50_ of 82.4 ± 0.85 and 16 ± 0.26 for the DPPH and TEAC assays, while for CUPRAC, FRAP, SNPAC, and EPR results were 219 ± 0.96 µM TE/100 mL, 290 ± 2.75 µM TE/ 100 mL, 679 ± 3.69 µM TE/100 mL, and 86.01 ± 0.77 integral intensity for the same types of sample. Together with the results obtained in the cytotoxicity assays, it is therefore shown that the aerial parts of this species present significant antioxidant capacity. Another study performed by our research team [15] highlights the antioxidant potential of the leaves of *B. alba*, but the present study is the first testing of the antioxidant capacity of the aerial parts of this species, and proving their promising potential as antioxidant agents. 

### 3.4. Cytotoxicity Assays and Anti-Plasmodial Assays

*Bryonia alba* is considered to be a toxic species, especially its roots, which have been cited to contain a toxic class of compounds, cucurbitacins [8]. Studies performed by our team [31] revealed that the aerial parts of the species do not contain these compounds. Moreover, the present study reveals the presence of significant levels of flavonoids. 

No cellular toxicity was observed for the tested samples at a concentration of 100 μg/mL or below, either on human cancerous cell lines and on human healthy cell lines. At the same time, anti-plasmodial tests showed no cellular toxicity on the parasitic strains used (Table 4). It could be therefore be established that the tested samples lack in vitro cellular toxicity. In this context, connections with the content of flavonoids could be established, and the potential of the aerial parts as antioxidant agents. 

### 3.5. Zebrafish Toxicity Assays

The zebrafish embryo acute toxicity test was used to assess the in vivo toxicity of the crude extract at different concentrations. It is a model used more and more by researchers worldwide as an alternative to animal testing [32], as zebrafish larvae represent early life stages of animals that are not protected until reaching 120 h after fertilization, when they are considered to be organisms with independent feeding [33]. The reasons for choosing this model for testing special toxicity of samples is related to the fast development of the embryos and also to their transparency, which allows observation of the changes that occur in different biological systems. Before the study, embryos were dechorionated, as chorions are the physical barrier that separate the embryo from the exterior. Thus, the contact of embryos with samples is facilitated. Various parameters were monitored at 24 hpf, 48 hpf, and 72 hpf, by comparing the exposed embryos to the control. Cardiovascular parameters such as heart rate and blood circulation, together with dermatological parameters and motility, were evaluated. At the same time, morphological changes in the development of the embryos were monitored. No significant change in these parameters were noticed during the experiment for tested samples (Table 5); all these parameters were found similar to those of the embryos not treated. These observations suggest the lack of toxicity in all observed systems, and therefore a lack of special toxicity. 

## 4. Conclusions

Analysis of flavonoids in the aerial parts of *Bryonia alba* L. showed the presence of four main flavonoids: lutonarin, saponarin, isoorientin, and isovitexin. The biological potential of these aerial parts was assessed by several in vitro and in vivo tests, which proved the lack of toxicity both to human cancerous and healthy cell lines and on parasitic strains. In this context, by using non-cellular assays, the antioxidant potential of these parts of the species could be established. The present study represents a promising premise for further tests that can support the use of these parts as medicinal products for different therapeutic purposes. 

## Figures and Tables

**Table 1:** The four flavonoids which are identified in the aerial parts of *Bryonia alba.*

Compound	Peak Number	Retention Time
Lutonarin	1	22.55 ± 0.27 min
Saponarin	2	24.78 ± 0.16 min
Isoorientin	3	25.98 ± 1.01 min
Isovitexin	4	28.87 min

**Table 2:** Quantification of the total phenolic content (TPC) and total flavonoid content (TFC) in the aerial parts of *Bryonia alba* L. (mg GAE or RE/100 g dvp).

Sample	TPC (mg GAE/100 g dvp)	TFC (mg RE/100 g dvp)
*Bryonia alba* aerial parts	3125 ± 0.31	2472 ± 0.94

Note: Values are the mean ± SD (*n* = 3).

**Table 3:** Assessment of the antioxidant capacity of the aerial parts of *Bryonia alba* L. (IC_50_ μg/mL or μM TE/100 mL).

Sample	*Bryonia alba* Aerial Parts
DPPH (IC_50_ * μg/mL)	99.8 ± 0.92
**CUPRAC (μM TE/100 mL)**	238 ± 2.24
**FRAP (μM TE/100 mL)**	217 ± 2.45
TEAC (IC_50_ ** μg/mL)	19.9 ± 0.89
**SNPAC (μM TE/100 mL)**	427 ± 2.46
EPR (Integral intensity ***)	401.96 ± 2.72

Positive controls: * quercetine: IC_50_ = 5.4 ± 0.32 μg/mL and BHT: IC_50_ = 15.6 ± 0.44 μg/mL; ** Trolox IC_50_ = 17.4 ± 0.26 μg/mL; *** DPPH Integral Intensity = 668.62 ± 0.18. Note: Values are the mean ± SD (*n* = 3).

**Table 4:** Assessment of the cytotoxicity and of the anti-plasmodial activity of the aerial parts of *Bryonia alba* L. (IC_50_ μg/mL).

Assay	*Bryonia alba* Aerial Parts	Artemisinin/Camptothecin
**Anti-plasmodial 3D7 (μg/mL)**	>50	0.009 ± 0.006
**Anti-plasmodial W2 (μg/mL)**	>50	0.017 ± 0.003
**Cytotoxic A549 (μg/mL)**	>50	0.167 ± 0.04
**Cytotoxic HeLa (μg/mL)**	>50	0.207 ± 0.27
**Cytotoxic WI38 (μg/mL)**	>50	0.076 ± 0.19

Note: Values are the mean ± SD (*n* = 3).

**Table 5:** Observation (40×) of zebrafish exposed to a concentration range of 0.1–100 μg/mL of the aerial parts of *Bryonia alba* compared to the control at 24 hpf, 48 hpf, and 72 hpf.

Sample	Zebrafish Assay Results 24 hpf	Zebrafish Assay Results 48 hpf	Zebrafish Assay 72 hpf
*Bryonia alba* aerial parts 100 μg/mL	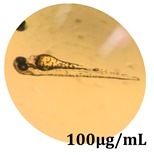	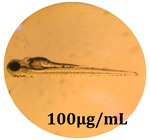	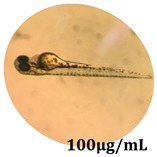
*Bryonia alba* aerial parts 10 μg/mL	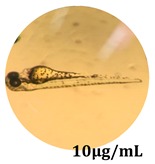	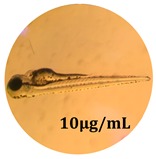	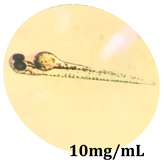
*Bryonia alba* aerial parts 1μg/mL	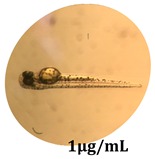	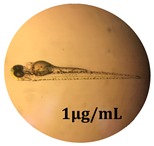	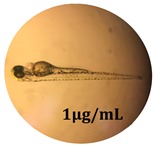
*Bryonia alba* aerial parts 0.1μg/mL	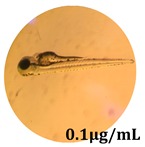	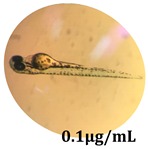	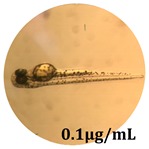
Non-treated zebrafish